# Hybrid‐type strigolactone analogues derived from auxins

**DOI:** 10.1002/ps.5553

**Published:** 2019-08-23

**Authors:** Daniel Blanco‐Ania, Jurgen J Mateman, Adéla Hýlová, Lukáš Spíchal, Luc M Debie, Binne Zwanenburg

**Affiliations:** ^1^ Institute for Molecules and Materials, Cluster of Organic Chemistry Radboud University Nijmegen the Netherlands; ^2^ Faculty of Science, Centre of the Region Haná for Biotechnological and Agricultural Research, Department of Chemical Biology and Genetics Palacký University Olomouc Czech Republic

**Keywords:** strigolactones, auxins, strigolactone analogues, suicidal germination

## Abstract

**BACKGROUND:**

Strigolactones (SLs) have a vast number of ecological implications because of the broad spectrum of their biological activities. Unfortunately, the limited availability of SLs restricts their applicability for the benefit of humanity and renders synthesis the only option for their production. However, the structural complexity of SLs impedes their economical synthesis, which is unfeasible on a large scale. Synthesis of SL analogues and mimics with a simpler structure, but with retention of bioactivity, is the solution to this problem.

**RESULTS:**

Here, we present eight new hybrid‐type SL analogues derived from auxin, synthesized via coupling of auxin ester [ethyl 2‐(1*H*‐indol‐3‐yl)acetate] and of ethyl 2‐phenylacetate with four D‐rings (mono‐, two di‐ and trimethylated). The new hybrid‐type SL analogues were bioassayed to assess the germination activity of seeds of the parasitic weeds *Striga hermonthica*, *Orobanche minor* and *Phelipanche ramosa* using the classical method of counting germinated seeds and a colorimetric method. The bioassays revealed that analogues with a natural monomethylated D‐ring had appreciable to good activity towards the three species and were the most active derivatives. By contrast, derivatives with the trimethylated D‐ring showed no activity. The dimethylated derivatives (2,4‐dimethyl and 3,4‐dimethyl) were slightly active, especially towards *P. ramosa*.

**CONCLUSIONS:**

New hybrid‐type analogues derived from auxins have been prepared. These analogues may be attractive as potential suicidal germination agents for parasitic weed control because of their ease of preparation and relevant bioactivity. © 2019 The Authors. *Pest Management Science* published by John Wiley & Sons Ltd on behalf of Society of Chemical Industry.

## Graphical abstract

Hybrid‐type strigolactone (SL) analogues were prepared from auxin ethyl ester in a two‐step operation. These SL analogues were bioassayed as germination agents for *Striga hermonthica*, *Pelipanche ramosa* and *Orobanche minor*.

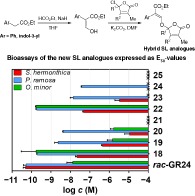

## 1 INTRODUCTION

**Figure 1:**
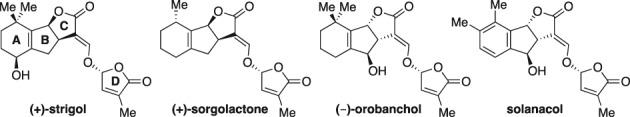
Naturally occurring strigolactones.

Strigolactones (SLs) constitute a group of new plant hormones that have received much interest in current plant biology.^1^, ^2^, ^3^, ^4^, ^5^, ^6^ SLs are present in many plants, particularly in root exudates.^7^ The first SL, strigol, was isolated in 1966 from the root exudate of cotton.^8^, ^9^, ^10^ Interestingly, strigol is an active germination stimulant for seeds of the parasitic weeds *Striga*, *Orobanche* and *Phelipanche* spp.^11^, ^12^ SLs invariably contain three annulated rings as the basic scaffold (the ABC ring system) connected to a butenolide (furanone; D‐ring) via an enol ether unit.^2^, ^13^ Most members of the SL family have been discovered since 1990. Elucidation of their structures was not easy and, as a consequence, several incorrect assignments appeared in the literature and were later corrected, for example (−)‐orobanchol,^14^, ^15^ alectrol^14^ and solanacol.^16^ At present, two families of SLs are known: one having stereochemistry as in (+)‐strigol and the other with stereochemistry as in (−)‐orobanchol (Fig. 1).^2^

New bio‐properties, such as a branching factor for arbuscular mycorrhizal fungi^17^ and the inhibition of bud outgrowth and shoot branching^18^, ^19^, ^20^, ^21^ were discovered at the beginning of this century and led to enormous interest in SLs. These findings led to their classification as a new class of plant hormones. Several reviews cover the current state of affairs concerning the chemistry and bio‐properties of SLs.^2^, ^3^, ^4^, ^5^, ^6^, ^22^, ^23^, ^24^

**Figure 2:**
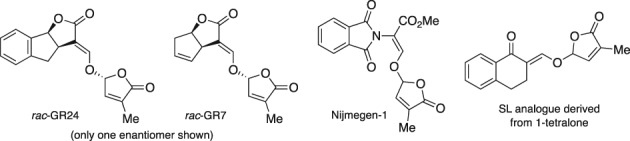
Important strigolactone analogues.

The availability of SLs is limited because levels of natural production are minute and their complete synthesis is quite laborious due to their complex structures.^25^, ^26^ Synthesis of simple bioactive analogues, having the same bioactiphore as natural SLs, has attracted considerable attention in recent years (Fig. 2). The ‘GR compounds’, such as GR24 and GR7, were the first series of such SL analogues.^27^ We have recently reported the use of some SL analogues in combating *Striga hermonthica* infestation in millet in sub‐Saharan Africa.^28^

**Figure 3:**
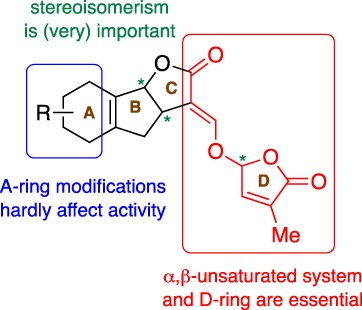
Working model for designing strigolactone analogues.

A model containing the features essential for activity was used to construct a large series of new analogues with excellent biological performance (Fig. 3).^2^, ^29^ Typical examples are Nijmegen‐1^30^ and analogues derived from simple ketones and keto enols (Fig. 2).^31^, ^32^

There is abundant evidence that detachment of the hydroxy D‐ring from SLs is the key step in triggering the germination of seeds of parasitic weeds.^5^, ^33^, ^34^ The part of the SL molecule that remains has, to our knowledge, no further function. Here, however, we construct an SL analogue having an ‘ABC’ scaffold that has a relevant bioactivity after detachment from the D‐ring. We call such compounds hybrid‐type SL analogues. Here, we use auxins, which are well‐known and widely used plant growth regulators, as functional ‘ABC’ scaffolds.^35^ Further evaluation to examine whether these SL analogues exhibit auxin‐like activity will form part of further research. Recently, Pereira *et al*.^36^ described the preparation of hybrid SL mimics derived from gibberellins, and we recently reported the synthesis of hybrid SL mimics from auxins.^37^ However, hybrid analogues having the same bioactiphore as natural SLs are new.

An additional incentive of the work described here is the preparation of a bioactive SL analogue from a readily available starting material in a simple synthetic operation.

## 2 MATERIALS AND METHODS

### 2.1 Synthesis

Reagents were obtained from commercial suppliers and were used without purification, except for ethyl formate which was distilled before use. Standard syringe techniques were used to transfer dry solvents and air‐ or moisture‐sensitive reagents. Reactions were followed, and *R*_F_ values were obtained using thin‐layer chromatography (TLC) on silica gel‐coated plates (Merck 60 F254) with the indicated solvent mixture. Detection was performed under ultraviolet (UV) light and by charring at ∼ 150 °C after dipping into a solution of either 2% anisaldehyde in ethanol/H_2_SO_4_, KMnO_4_ or ninhydrin. Nuclear magnetic resonance (^1^H and ^13^C NMR) spectra were recorded at 298 K on a Varian Inova 400 (400 MHz), Bruker Avance III 400 MHz or Bruker Avance III 500 MHz spectrometer in the solvent indicated. Chemical shifts are given in parts per million (ppm) with respect to tetramethylsilane (0.00 ppm) as the internal standard for ^1^H NMR and CDCl_3_ (77.16 ppm) as the internal standard for ^13^C NMR. Coupling constants are reported as *J* values in hertz (Hz). High‐resolution mass spectra (HRMS) were recorded with JEOL AccuTOF mass spectrometer. Column or flash chromatography was carried out using ACROS silica gel (0.035–0.070 mm and 60 Å pore diameter).

#### 2.1.1 *5‐Hydroxy‐3‐methylfuran‐2(5*H*)‐one (* ***5*** *)*

 


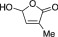
Method 1: Methylmalonic acid (1.0 g, 8.47 mmol, 1 eq.) and phenylboronic acid (1.1 g, 8.89 mmol, 1.05 eq.) were dissolved in water (10 mL) and a 40% aqueous solution of glyoxal (1.46 mL, 12.7 mmol, 1.5 eq.) was added. The mixture was stirred and heated under reflux (100 °C) for 16 h. The mixture was then allowed to cool to 20 °C, and was saturated with solid NaCl. The mixture was extracted with EtOAc (3 × 20 mL). The combined organic extracts were dried over Na_2_SO_4_ and concentrated *in vacuo*. The crude product was purified by flash column chromatography (silica, 40% EtOAc in heptane) to yield **5** (630 mg, 65%) as a yellow solid.

Method 2: A 40% solution of glyoxal in water (14 mL, 121.0 mmol, 1.4 eq.) and 20 drops of concentrated H_2_SO_4_ were added to a solution of methylmalonic acid (10.0 g, 84.7 mmol, 1 eq.) in water (100 mL). The mixture was heated under reflux (100 °C) for 18 h. The solution was then allowed to cool to 20 °C and was saturated with solid NaCl (40 g). The solution was extracted with EtOAc (3 × 60 mL). The combined organic extracts were dried over MgSO_4_ and concentrated *in vacuo*. The crude product was purified by flash column chromatography (silica, 40% EtOAc in heptane) to yield **5** (4.17 g, 43%) as a yellow solid. ^1^H NMR (400 MHz, CDCl_3_) δ 6.88 (p, *J* = 1.6 Hz, 1 H), 6.08 (s, 1 H), 3.23 (br s, 1 H), 1.96 (t, *J* = 1.6 Hz, 3 H).

#### 2.1.2 *5‐Hydroxy‐3,5‐dimethylfuran‐2(5*H*)‐one (* ***6*** *)*

 


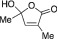
Methylmalonic acid (1.0 g, 8.47 mmol, 1 eq.) and phenylboronic acid (1.1 g, 8.89 mmol, 1.05 eq.) were dissolved in water (10 mL) and a 40% aqueous solution of pyruvaldehyde (0.8 mL, 12.7 mmol, 1.5 eq.) was added. The mixture was stirred and heated under reflux (100 °C) for 16 h. The mixture was then allowed to cool to 20 °C, and was saturated with solid NaCl. The mixture was extracted with EtOAc (3 × 20 mL). The combined organic extracts were dried over Na_2_SO_4_ and concentrated *in vacuo*. The crude product was purified by flash column chromatography (silica, 40% EtOAc in heptane) to yield **6** (605 mg, 55%) as a yellow solid. This solid was further purified by recrystallization from DCM/heptane (1:1) to yield **6** as white needles. ^1^H NMR (400 MHz, CDCl_3_) δ 6.86 (q, *J* = 1.7 Hz, 1 H), 3.04 (br s, 1 H), 1.92 (d, *J* = 1.7 Hz, 3 H), 1.69 (s, 3 H). ^13^C NMR (101 MHz, CDCl_3_) δ 171.4, 147.4, 131.8, 104.0, 24.8, 10.4.

#### 2.1.3 *5‐Hydroxy‐3,4‐dimethylfuran‐2(5*H*)‐one (* ***7*** *)*

 


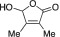
A solution of 2,3‐dimethylmaleic anhydride (1.0 g, 7.9 mmol, 1 eq.) in dry tetrahydrofuran (THF; 25 mL) was stirred and cooled to −15 °C. To this solution, a solution of lithium hydrido[tris(2‐methylpropan‐2‐olato)]aluminate (Li[AlH(O^*t*^Bu)_3_]; 2.8 g, 10.9 mmol, 1.4 eq.) in dry THF (15 mL) was added dropwise over 10 min. The mixture was stirred at −15 °C for 1 h, and then at 20 °C for 1 h. The reaction was quenched by adding 2 m aqueous HCl (40 mL), and then extracted with EtOAc (3 × 40 mL). The combined organic extracts were washed with brine, dried over MgSO_4_, and concentrated *in vacuo* to yield **7** (560 mg, 55%) as a white solid that was used without further purification. ^1^H NMR (400 MHz, CDCl_3_) δ 5.87 (s, 1 H), 3.57 (br s, 1 H), 2.01 (p, *J* = 1.1 Hz, 3 H), 1.84 (p, *J* = 1.2 Hz, 3 H).

#### 2.1.4 *5‐Hydroxy‐3,4,5‐trimethylfuran‐2(5*H*)‐one (* ***8*** *)*

 


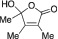
Method 1: Methylmalonic acid (1.0 g, 8.47 mmol, 1 eq.) and phenylboronic acid (1.1 g, 8.89 mmol, 1.05 eq.) were dissolved in water (10 mL) and butane‐2,3‐dione (1.1 mL, 12.7 mmol, 1.5 eq.) was added. The mixture was stirred and heated under reflux (100 °C) for 16 h. The mixture was then allowed to cool to 20 °C, and was saturated with solid NaCl. The mixture was extracted with EtOAc (3 × 20 mL). The combined organic extracts were dried over Na_2_SO_4_ and concentrated *in vacuo*. The crude product was purified by flash column chromatography (silica, 30% EtOAc in heptane) to yield **8** (545 mg, 46%) as a yellow solid.

Method 2: A solution of 2,3‐dimethylmaleic anhydride (1.0 g, 7.9 mmol, 1 eq.) in dry THF (50 mL) was stirred and cooled to −78 °C under a nitrogen atmosphere. A 1.6 m solution of methyllithium in Et_2_O (5.0 mL, 8.0 mmol, 1 eq.) was added dropwise over 15 min and the resulting mixture was stirred at −78 °C for 15 min. The reaction was quenched by adding a saturated aqueous solution of ammonium chloride (NH_4_Cl) (20 mL), and the mixture was extracted with EtOAc (3 × 30 mL). The combined organic extracts were washed with brine, dried over MgSO_4_ and concentrated *in vacuo*. The residue was subjected to flash column chromatography (silica, 30% EtOAc in heptane) to yield **8** (375 mg, 33%) as a white solid. ^1^H NMR (400 MHz, CDCl_3_) δ 4.55 (br s, 1 H), 1.97 (q, *J* = 1.1 Hz, 3 H), 1.78 (q, *J* = 1.1 Hz, 3 H), 1.62 (s, 3 H).

##### 2.1.4.1 *General procedure for the synthesis of chloro furanones (* ***9*** *–* ***12*** *)*

A solution of thionyl chloride (6 mmol, 1.5 eq.) and one drop of *N*,*N*‐dimethylformamide (DMF) in CH_2_Cl_2_ (3 mL) was stirred and heated under reflux (40 °C). Hydroxy furanone (**5**–**8**, 4 mmol, 1 eq.) was dissolved in CH_2_Cl_2_ (3 mL) and then added dropwise to the refluxing solution. The resulting mixture was refluxed for 2 h. After the mixture was cooled to 20 °C, the mixture was diluted with CH_2_Cl_2_ (20 mL) and then poured into a cooled (0 °C) solution of saturated aqueous NaHCO_3_ (25 mL) and stirred until gas evolution had stopped. The aqueous phase was extracted with CH_2_Cl_2_ (3 × 25 mL). The combined organic extracts were washed with brine, dried over Na_2_SO_4_ and concentrated *in vacuo* to give the crude product as a pale yellow oil. The crude product was distilled using a Kugelrohr apparatus (18 mbar, 150 °C) to give the pure chloro furanone (**9**–**12**) as a colourless liquid.

#### 2.1.5 *5‐Chloro‐3‐methylfuran‐2(5*H*)‐one (* ***9*** *)*

 


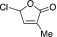
Yield: 349 mg (48%). ^1^H NMR (400 MHz, CDCl_3_) δ 7.08 (p, *J* = 1.6 Hz, 1 H), 6.54 (p, *J* = 1.5 Hz, 1 H), 2.01 (t, *J* = 1.6 Hz, 3 H). ^13^C NMR (101 MHz, CDCl_3_) δ 170.8, 145.9, 131.9, 85.2, 10.5.

Method 2: In a flame‐dried flask under nitrogen atmosphere, CCl_4_ (26.9 g, 16.9 mL, 175.1 mmol, 2.0 eq.) and triphenylphosphane (Ph_3_P; 34.5 g, 131.5 mmol, 1.5 eq.) were added sequentially to a solution of **5** (10.0 g, 87.6 mmol, 1.0 eq.) in dry THF (300 mL). The reaction was stirred at 60 °C for 3 h. The reaction mixture was then cooled to 23 °C and concentrated *in vacuo*. The residue was stirred in Et_2_O/pentane (1:1; 250 mL) until triphenylphosphane oxide (Ph_3_PO) precipitated. The suspension was filtered; the filtrate was then flushed through a pad of silica gel and concentrated *in vacuo* to yield **9** (9.55 g, 82%) as a slightly yellow oil.

#### 2.1.6 *5‐Chloro‐3,5‐dimethylfuran‐2(5*H*)‐one (* ***10*** *)*

 


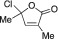
Yield: 572 mg (80%). ^1^H NMR (500 MHz, CDCl_3_) δ 7.11 (q, *J* = 1.7 Hz, 1 H), 2.02 (s, 3 H), 1.98 (d, *J* = 1.7 Hz, 3 H). ^13^C NMR (126 MHz, CDCl_3_) δ 170.5, 150.1, 129.6, 97.9, 29.3, 10.2.

#### 2.1.7 *5‐Chloro‐3,4‐dimethylfuran‐2(5*H*)‐one (* ***11*** *)*

 


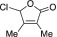
Yield: 590 mg (65%). ^1^H NMR (400 MHz, CDCl_3_) δ 6.39–6.35 (m, 1 H), 2.08 (p, *J* = 1.1 Hz, 3 H), 1.89 (p, *J* = 1.2 Hz, 3 H). ^13^C NMR (101 MHz, CDCl_3_) δ 171.1, 156.8, 125.7, 88.2, 11.8, 8.6.

#### 2.1.8 *5‐Chloro‐3,4,5‐trimethylfuran‐2(5*H*)‐one (* ***12*** *)*

 


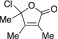
Yield: 328 mg (94%). ^1^H NMR (400 MHz, CDCl_3_) δ 2.08 (q, *J* = 1.1 Hz, 3 H), 1.94 (s, 3 H), 1.86 (q, *J* = 1.1 Hz, 3 H). ^13^C NMR (101 MHz, CDCl_3_) δ 170.4, 160.0, 123.8, 100.4, 28.0, 10.8, 8.6.

#### 2.1.9 *Ethyl 3‐oxo‐2‐phenylpropanoate (* ***16a*** *) and ethyl (*Z*)‐3‐hydroxy‐2‐phenylacrylate (* ***16b*** *)*

 


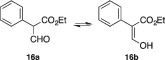
Sodium hydride (2.0 g, 48.7 mmol, 4 eq.; 60% dispersion in mineral oil) was washed with pentane (3 × 10 mL) under a nitrogen atmosphere. Dry THF (40 mL) was added and the mixture was stirred and cooled to 0 °C. Ethyl phenylacetate (2.0 g, 12.2 mmol, 1 eq.) was dissolved in ethyl formate (5 mL) and added dropwise to the stirred solution. The reaction mixture was stirred at 20 °C for 24 h in total. Additional amounts of ethyl formate (2.5 mL per time) were added after 6 and 16 h. The reaction was quenched by adding ethanol (EtOH; 15 mL) slowly, followed by the addition of a 50% (v/v) aqueous solution of acetic acid (AcOH; 20 mL). Diethyl ether was added until the aqueous and organic phases separated. The separated aqueous layer was extracted with Et_2_O (3 × 25 mL). The combined organic extracts were washed with brine, dried over MgSO_4_ and concentrated *in vacuo* to yield **16a**/**16b** (2.27 g, 97%) as a yellow oil that was used without further purification. ^1^H NMR (400 MHz, CDCl_3_) δ 12.12 (d, *J* = 12.6 Hz, 1 H), 7.30 (d, *J* = 12.6 Hz, 1 H), 7.37–7.23 (m, 5 H), 4.30 (q, *J* = 7.1 Hz, 2 H), 1.29 (t, *J* = 7.1 Hz, 3 H).

(Tautomers **16a** and **16b** are both observed in a 1:12 ratio in the ^1^H NMR spectrum. The reported signals belong to the major product **16b**.)

#### 2.1.10 *Ethyl 2‐(1*H*‐indol‐3‐yl)‐3‐oxopropanoate (* ***17a*** *) and ethyl (*Z*)‐3‐hydroxy‐2‐(1*H*‐indol‐3‐yl)acrylate (* ***17b*** *)*

 


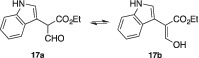
Sodium hydride (1.0 g, 25 mmol, 5 eq.; 60% dispersion in mineral oil) was washed with pentane (3 × 5 mL) under a nitrogen atmosphere. Dry THF (20 mL) was added and the mixture was stirred and cooled to 0 °C. Ethyl 2‐(1*H*‐indol‐3‐yl)acetate (1.0 g, 4.92 mmol, 1 eq.) was dissolved in ethyl formate (4 mL) and added dropwise to the stirred solution. The reaction mixture was stirred at 20 °C for 36 h in total. Additional amounts of ethyl formate (2 mL per time) were added after 16 and 24 h. The reaction was quenched by adding EtOH (15 mL) slowly, followed by the addition of a 50% (v/v) aqueous solution of AcOH (20 mL). Diethyl ether was added until the aqueous and organic phases separated. The separated aqueous layer was extracted with Et_2_O (3 × 25 mL). The combined organic extracts were washed with a solution of saturated aqueous NaHCO_3_, dried over MgSO_4_ and concentrated *in vacuo*, to give **17a**/**17b** (1.03 g, 90%) as a grey solid that was used without further purification. ^1^H NMR (400 MHz, CDCl_3_) δ 12.13 (d, *J* = 12.6 Hz, 1 H), 8.12 (br s, 1 H), 7.58–7.54 (m, 1 H), 7.42 (d, *J* = 12.5 Hz, 1 H), 7.38 (dt, *J* = 8.1, 1.0 Hz, 1 H), 7.21 (ddd, *J* = 8.1, 7.1, 1.3 Hz, 1 H), 7.16–7.11 (m, 2 H), 4.29 (q, *J* = 7.1 Hz, 2 H), 1.26 (t, *J* = 7.1 Hz, 3 H). ^13^C NMR (101 MHz, CDCl_3_) δ 172.3, 162.9, 135.9, 127.2, 123.2, 122.2, 119.8, 119. 8, 111.2, 109.5, 100.6, 60.8, 14.2.

(Tautomers **17a** and **17b** are both observed in a 1:9 ratio in the ^1^H NMR spectrum. The reported signals belong to the major product **17b**.)

##### 2.1.10.1 *General procedure for the synthesis of strigolactone hybrids* ***18*** *–* ***25***

A flame‐dried Schlenk flask was loaded with hydroxymethylidene scaffold (**16** or **17**; 1 mmol, 1 eq.) and potassium carbonate (1.1 eq.), and was dried on a vacuum pump for 2 h. The flask was then filled with nitrogen and cooled on an ice bath (0 °C). Dry DMF (4 mL) was added and the mixture was stirred for 30 min on the ice bath. The stirred mixture was then cooled to −40 °C and a solution of chloro furanone (**9**–**12**, 1.2 eq.) in DMF (1 mL) was added dropwise to the stirred solution. The cooling bath was removed and the reaction mixture was stirred at 20 °C for 65 h. The reaction was quenched with water (5 mL), EtOAc (5 mL) was added and the organic phase was washed with ice‐cold brine (4 × 5 mL). The organic phase was separated and the combined washings were back extracted with EtOAc (10 mL). This extract was washed again with ice‐cold brine (4 × 5 mL). The organic extracts were combined, dried over MgSO_4_ and concentrated *in vacuo*. The crude product was purified by flash column chromatography (silica, 30% EtOAc in heptane).

#### 2.1.11 *Ethyl (*E*)‐3‐[(4‐methyl‐5‐oxo‐2,5‐dihydrofuran‐2‐yl)oxy]‐2‐ phenylacrylate (* ***18*** *)*

 


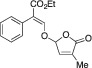
Yellow oil. Yield: 213 mg (71%). ^1^H NMR (400 MHz, CDCl_3_) δ 7.73 (s, 1 H), 7.37–7.23 (m, 5 H), 6.81 (q, *J* = 1.6 Hz, 1 H), 6.10 (q, *J* = 1.4 Hz, 1 H), 4.22 (q, *J* = 7.1 Hz, 2 H), 1.95–1.91 (m, 3 H), 1.27 (t, *J* = 7.1 Hz, 3 H). ^13^C NMR (101 MHz, CDCl_3_) δ 170.6, 166.8, 152.6, 141.5, 135.3, 131.8, 130.1, 127.8, 127.5, 115.8, 100.6, 60.8, 14.3, 10.6. HRMS [ESI^+^ (*m*/*z*)] calcd for (C_16_H_16_O_5_ + Na)^+^ 311.08954, found 311.08931.

#### 2.1.12 *Ethyl (*E*)‐3‐[(2,4‐dimethyl‐5‐oxo‐2,5‐dihydrofuran‐2‐yl)oxy]‐2‐phenylacrylate (* ***19*** *)*

 


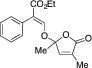
White solid. Yield: 206 mg (70%). ^1^H NMR (400 MHz, CDCl_3_) δ 7.54 (s, 1 H), 7.38–7.26 (m, 5 H), 6.83 (q, *J* = 1.7 Hz, 1 H), 4.21 (q, *J* = 7.1 Hz, 2 H), 2.00 (d, *J* = 1.7 Hz, 3 H), 1.72 (s, 3 H), 1.26 (t, *J* = 7.1 Hz, 3 H). ^13^C NMR (101 MHz, CDCl_3_) δ 170.3, 166.9, 148.7, 145.9, 133.8, 132.0, 130.1, 127.7, 127.3, 115.7, 106.3, 60.7, 23.5, 14.3, 10.5. HRMS [ESI^+^ (*m*/*z*)] calcd for (C_17_H_18_O_5_ + Na)^+^ 325.10519, found 325.10484.

#### 2.1.13 *Ethyl (*E*)‐3‐[(3,4‐dimethyl‐5‐oxo‐2,5‐dihydrofuran‐2‐yl)oxy]‐2‐phenylacrylate (* ***20*** *)*

 


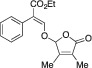
Yellow oil. Yield: 324 mg (86%). ^1^H NMR (400 MHz, CDCl_3_) δ 7.73 (s, 1 H), 7.41–7.18 (m, 5 H), 5.94 (s, 1 H), 4.22 (q, *J* = 7.1 Hz, 2 H), 1.90 (s, 3 H), 1.81 (s, 3 H), 1.27 (t, *J* = 7.1 Hz, 3 H). ^13^C NMR (101 MHz, CDCl_3_) δ 171.0, 166.8, 152.9, 152.6, 131.8, 130.1, 127.8, 127.8, 127.5, 115.9, 102.1, 60.8, 14.3, 11.4, 8.5. HRMS [ESI^+^ (*m*/*z*)] calcd for (C_17_H_18_O_5_ + Na)^+^ 325.10519, found 325.10495.

#### 2.1.14 *Ethyl (*E*)‐2‐phenyl‐3‐[(2,3,4‐trimethyl‐5‐oxo‐2,5‐dihydrofuran‐2‐yl)oxy]acrylate (* ***21*** *)*

 


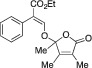
Yellow solid. Yield: 340 mg (86%). ^1^H NMR (400 MHz, CDCl_3_) δ 7.48 (s, 1 H), 7.40–7.29 (m, 5 H), 4.25–4.17 (m, 2 H), 1.91 (s, 3 H), 1.91 (s, 3 H), 1.69 (s, 3 H), 1.28 (t, *J* = 7.1 Hz, 3 H). ^13^C NMR (101 MHz, CDCl_3_) δ 169.4, 165.9, 155.5, 147.5, 131.0, 129.0, 126.6, 126.3, 125.7, 114.8, 106.4, 59.6, 21.2, 13.2, 9.5, 7.5. HRMS [ESI^+^ (*m*/*z*)] calcd for (C_18_H_20_O_5_ + Na)^+^ 339.12084, found 339.12080.

#### 2.1.15 *Ethyl (*E*)‐2‐(1*H*‐indol‐3‐yl)‐3‐[(4‐methyl‐5‐oxo‐2,5‐dihydrofuran‐2‐yl)oxy]acrylate (* ***22*** *)*

 


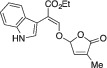
Orange solid. Yield: 121 mg (57%). ^1^H NMR (400 MHz, CDCl_3_) δ 8.31 (br s, 1 H), 7.79 (s, 1 H), 7.43–7.39 (m, 1 H), 7.32 (dt, *J* = 8.1, 1.0 Hz, 1 H), 7.27 (d, *J* = 2.6 Hz, 1 H), 7.16 (ddd, *J* = 8.2, 7.6, 1.3 Hz, 1 H), 7.09 (ddd, *J* = 8.0, 7.0, 1.1 Hz, 1 H), 6.78 (p, *J* = 1.6 Hz, 1 H), 6.15 (p, *J* = 1.4 Hz, 1 H), 4.27 (q, *J* = 7.1 Hz, 2 H), 1.91 (t, *J* = 1.6 Hz, 3 H), 1.30 (t, *J* = 7.1 Hz, 3 H). ^13^C NMR (101 MHz, CDCl_3_) δ 170.7, 167.3, 151.7, 141.5, 135.6, 135.2, 126.3, 125.3, 121.9, 120.8, 119.7, 111.2, 109.6, 106.7, 100.5, 60.9, 14.4, 10.6. HRMS [ESI^+^ (*m*/*z*)] calcd for (C_18_H_17_NO_5_ + Na)^+^ 350.10044, found 350.09998.

#### 2.1.16 *Ethyl (*E*)‐3‐[(2,4‐dimethyl‐5‐oxo‐2,5‐dihydrofuran‐2‐yl)oxy]‐2‐(1*H*‐indol‐3‐yl)acrylate (* ***23*** *)*

 


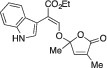
Yellow solid. Yield: 89 mg (46%). ^1^H NMR (400 MHz, CDCl_3_) δ 8.26 (br s, 1 H), 7.58 (s, 1 H), 7.46–7.43 (m, 1 H), 7.38 (dt, *J* = 8.1, 1.0 Hz, 1 H), 7.34 (d, *J* = 2.6 Hz, 1 H), 7.19 (ddd, *J* = 8.2, 7.0, 1.3 Hz, 1 H), 7.11 (ddd, *J* = 8.0, 7.0, 1.1 Hz, 1 H), 6.84 (q, *J* = 1.7 Hz, 1 H), 4.28–4.21 (m, 2 H), 2.00 (d, *J* = 1.7 Hz, 3 H), 1.71 (s, 3 H), 1.27 (t, *J* = 7.1 Hz, 3 H). ^13^C NMR (101 MHz, CDCl_3_) δ 170.4, 167.5, 147.9, 146.0, 135.6, 133.9, 126.5, 125.2, 121.9, 121.1, 119.5, 111.1, 109.2, 107.3, 106.3, 60.7, 23.7, 14.3, 10.5. HRMS [ESI^+^ (*m*/*z*)] calcd for (C_19_H_19_NO_5_ + Na)^+^ 364.11609, found 364.11565.

#### 2.1.17 *Ethyl (*E*)‐3‐[(3,4‐dimethyl‐5‐oxo‐2,5‐dihydrofuran‐2‐yl)oxy]‐2‐(1*H*‐indol‐3‐yl)acrylate (* ***24*** *)*

 


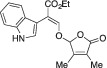
Orange solid. Yield: 64 mg (29%). ^1^H NMR (400 MHz, CDCl_3_) δ 8.27 (br s, 1 H), 7.80 (s, 1 H), 7.47–7.43 (m, 1 H), 7.37 (dt, *J* = 8.1, 0.9 Hz, 1 H), 7.33 (d, *J* = 2.6 Hz, 1 H), 7.18 (ddd, *J* = 8.2, 7.1, 1.1 Hz, 1 H), 7.09 (ddd, *J* = 8.1, 7.1, 1.1 Hz, 1 H), 6.00 (sept, *J* = 0.9 Hz, 1 H), 4.27 (q, *J* = 7.1 Hz, 2 H), 1.90 (p, *J* = 1.0 Hz, 3 H), 1.81 (p, *J* = 1.2 Hz, 3 H), 1.30 (t, *J* = 7.1 Hz, 4 H). ^13^C NMR (101 MHz, CDCl_3_) δ 171.1, 167.3, 152.8, 151.8, 135.5, 127.8, 126.4, 125.2, 121.9, 121.0, 119.6, 111.1, 109.5, 107.0, 102.2, 60.8, 14.4, 11.6, 8.5. HRMS [ESI^+^ (*m*/*z*)] calcd for (C_19_H_19_NO_5_ + Na)^+^ 364.11609, found 364.11574.

#### 2.1.18 *Ethyl (*E*)‐2‐(1*H*‐indol‐3‐yl)‐3‐[(2,3,4‐trimethyl‐5‐oxo‐2,5‐dihydrofuran‐2‐yl)oxy]‐acrylate (* ***25*** *)*

 


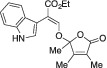
Orange solid. Yield: 74 mg (42%). ^1^H NMR (400 MHz, CDCl_3_) δ 8.30 (br s, 1 H), 7.51 (s, 1 H), 7.48–7.44 (m, 1 H), 7.36 (dt, *J* = 8.1, 1.0 Hz, 1 H), 7.32 (d, *J* = 2.6 Hz, 1 H), 7.18 (ddd, *J* = 8.2, 7.0, 1.3 Hz, 1 H), 7.10 (ddd, *J* = 8.1, 7.0, 1.1 Hz, 1 H), 4.29–4.18 (m, 2 H), 1.87 (q, *J* = 1.2 Hz, 3 H), 1.83 (q, *J* = 1.2 Hz, 3 H), 1.67 (s, 3 H), 1.27 (t, *J* = 7.1 Hz, 3 H). ^13^C NMR (101 MHz, CDCl_3_) δ 170.6, 167.5, 156.6, 147.6, 135.6, 126.8, 126.5, 125.2, 121.8, 121.2, 119.5, 111.1, 109.4, 107.4, 107.2, 60.7, 22.4, 14.3, 10.6, 8.6. HRMS [ESI^+^ (*m*/*z*)] calcd for (C_20_H_21_NO_5_ + Na)^+^ 378.13174, found 378.13106.

### 2.2 Bioassays

Seeds of parasitic weeds were sterilized in a solution of 2% (v/v) sodium hypochorite and 1% (v/v) Triton X‐100 for 6.5 min and washed with MilliQ water. Considering classical germination method,^38^ glass fibre filter paper discs (10 mm, Grade GF/D, Whatman, GE Healthcare, UK) were placed in a six‐well plate (three per well) and ∼ 25 seeds were spread onto each. MilliQ water was added carefully (1.6 mL/well), plates were sealed and conditioned (i.e. warm stratified) in the dark (for details see Table 1). Consequently, discs were dried on a filter paper for 30 min, placed back into plates and germination was induced with compound solutions (100 μL per disc). Six days later, the assay was evaluated by counting under binocular and calculating percentage of germinated seeds.

**Table 1:** Conditioning period and temperature for germination of parasitic weed seeds

Species	Collection	Temperature (°C)	Conditioning (days)
*Striga hermonthica*	Sudan, 2007	27	7
*Phelipanche ramosa*	St. Martin de Bossenay, France, 2012, host *Cannabis sativa*	21	7
*Orobanche minor*	Porto Cedro, Sardinia, 2011, host *Asteraceae*	21	14

For the colorimetric method,^39^ seeds were sterilized as described above and incubated in 1 mm Hepes, pH 7.5, and 0.1% PPM (Plant Preservative Mixture, Diagnovation Technologies, NY, USA) in 50 mL falcon tubes in the dark. Distribution into a 96‐well plate and stimulation with solutions of tested compounds followed after a conditioning period. MTT solution (5 g/L, 10 μL per well) was added after 4 days of germination. After 24 h, emerged formazan salts were solubilized by 100 μL of lyse buffer (10% Triton X‐100 and 0.04% HCl in propan‐2‐ol). Absorbances at 570 and 690 nm were measured after 24 h and differences (*A*_570_–*A*_690_) calculated.

Dose–response analysis was performed in GraphPad Prism 5.0 and results expressed as EC_50_ (half‐maximal concentration).

## 3 RESULTS AND DISCUSSION

### 3.1 Synthesis

**Scheme 1:**
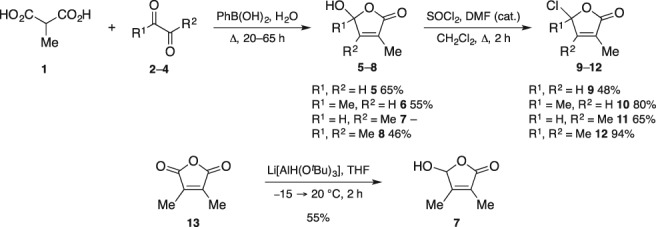
Synthesis of hydroxy furanones **5**–**8** and chloro furanones **9**–**12**.

The synthesis of the new hybrid‐type SL analogues from auxins commenced with the synthesis of four hydroxy furanones (**5**–**8**; Scheme 1): monomethylated **5**, dimethylated **6** and **7**, and trimethylated **8**. The activity of SL analogues with polymethylated D‐rings is sometimes comparable with the derivatives with the natural monomethylated D‐ring^40^ and at times, the corresponding SL analogues might be easier (and cheaper on large scale) to synthesize. We developed a new one‐step procedure for the synthesis of hydroxy furanones **5**, **6** and **8**.^41^ Methylmalonic acid (**1**) was condensed with glyoxal (**2**), pyruvaldehyde (**3**) and butane‐2,3‐dione (**4**) with the aid of phenylboronic acid in refluxing water to yield hydroxy furanones **5**, **6** and **8** in 65, 55 and 46% yields, respectively. Alternatively, hydroxy furanone **5** was also synthesized by condensation of methylmalonic acid (**1**) with glyoxal (**2**) catalysed by sulfuric acid^42^, ^43^ and hydroxy furanone **8** was synthesized from dimethylmaleic anhydride and MeLi^44^ with yields (depending on the scale) comparable with the previous method. Hydroxy furanone **7** was synthesized by reduction of dimethylmaleic anhydride (**13**) with Li[AlH(O^*t*^Bu)_3_] in 55% yield (Scheme 1).^44^

The coupling of SL analogues with the D‐ring is commonly achieved with bromo furanones, however, the coupling with the chloro furanones is less capricious. Thus, chloro furanones **9**–**12** were then synthesized by reaction of hydroxy furanones **5**–**8** with thionyl chloride in fair to excellent yields (48–94%) depending on the substitution (Scheme 1). The more substituted the reactive carbon is, the higher the yield obtained, because the rate of the competing formation of the side product (D‐ring)_2_ O is then slowed. Compound **9** was also synthesized in 82% yield using the Appel reaction (CCl_4_, Ph_3_P, THF, 60 °C, 3 h).

**Scheme 2:**
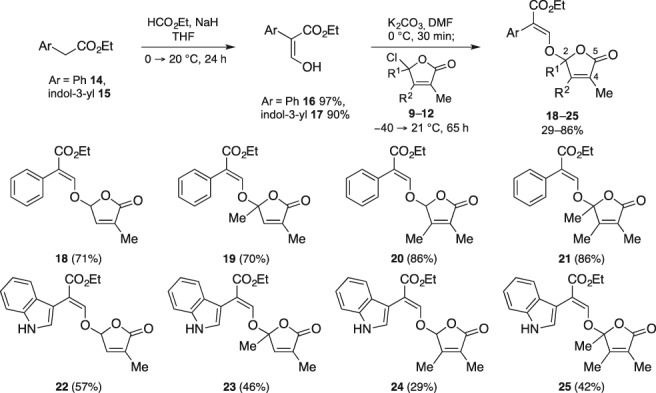
Synthesis of hybrid‐type strigolactone analogues **18**–**25**.

Finally, the hybrid‐type SL analogues were readily prepared via the regular two‐step sequence utilized at the end of every state‐of‐the‐art total synthesis of SLs: formylation of the scaffolds followed by coupling of the D‐ring. Consequently, the commercially available ethyl ester auxins **14** and **15** were formylated using ethyl formate in the present of a base (NaH) in excellent yields and the resulting enols **16** and **17**, respectively, were then coupled with chloro furanones **9**–**12** using potassium carbonate in DMF to afford hybrid‐type SL analogues **18**–**25** in poor to very good yields (Scheme 2). In all cases, the yields of the products derived from auxin derivative **14** (**18**–**21**) were higher than those of the counterparts of auxin derivative **15** (**22**–**25**).

All these syntheses fulfil the criterion of an easy synthetic operation: only two synthetic steps are needed (excluding formation of the always necessary Cl–D‐rings) from cheap commercially available compounds.

### 3.2 Bioassays

**Figure 4:**
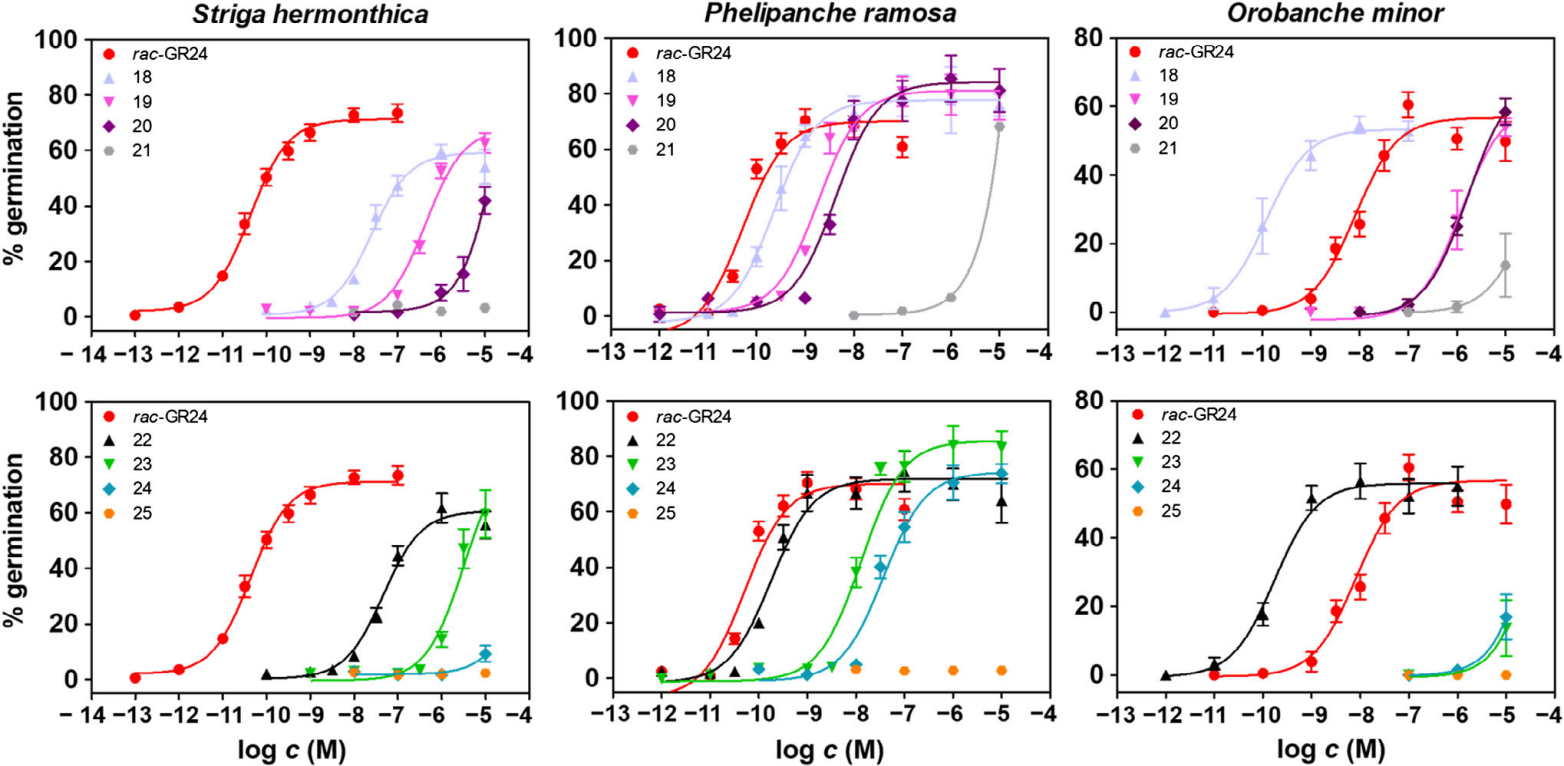
Dose–response curves of hybrid strigolactone analogues tested for germination stimulatory activity of parasitic weed seeds. Data represent averages with standard error (*n* ≥ 2).

Hybrid‐type SL analogues **18**–**25** were bioassayed for germination of seeds of the parasitic weeds *Striga hermonthica*, *Phelipanche ramosa* and *Orobanche minor*. The number of germinated seeds was counted in the traditional manner^38^ and also analysed using a colorimetric method.^39^ We showed earlier that this colorimetric method gives the same results as the classical seed counting method.^37^ Some of the compounds showed a remarkably high activity. The compounds were tested in a wide concentration range and dose–response curves were generated in GraphPad Prism 5.0 (Fig. 4).

**Figure 5:**
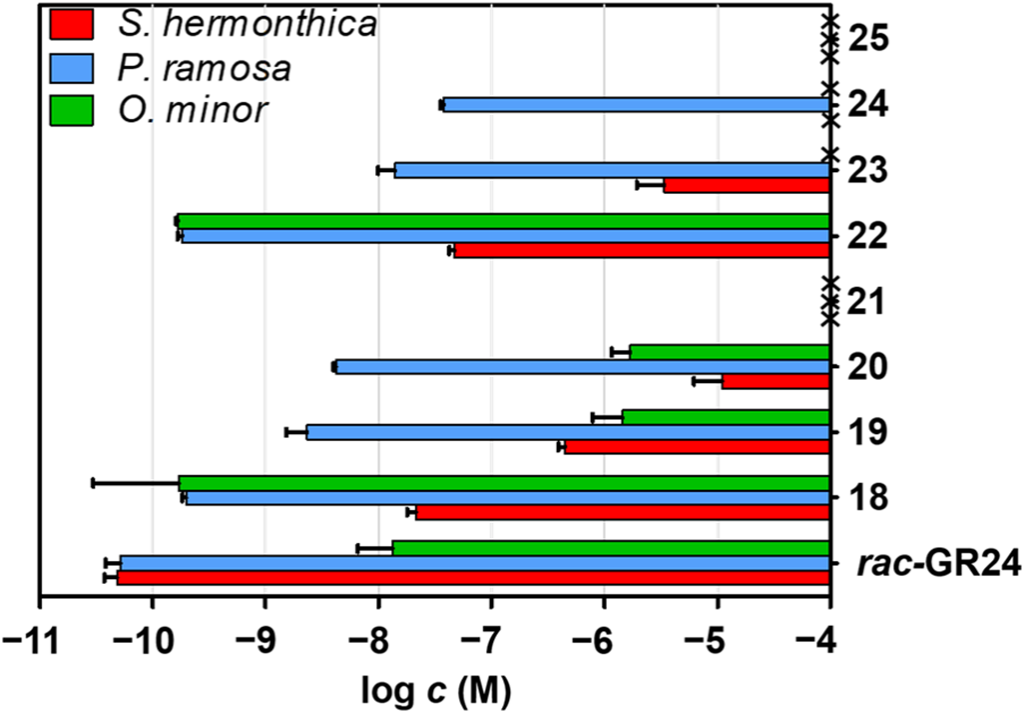
Bioactivity of new strigolactone hybrids expressed as EC_50_ values. Crosses indicate no stimulatory activity. Data represent averages with standard error (*n* ≥ 2).

A non‐linear regression was employed to compute EC_50_ values from dose–response curves. Graphical presentation of the EC_50_ values is given in Fig. 5.

Hybrids **18** and **22**, with the monomethylated natural D‐ring, presented similar results and exhibited the best activity towards the three species. Their activities towards *P. ramosa* were comparable with that of GR24 and those towards *O. minor* were considerably higher. By contrast, hybrids **21** and **25** (with trimethylated D‐rings) did not present any activity towards any of the three species. Dimethylated hybrid SL analogues derived from ethyl ester auxin **14** (**19** and **20**) were more active towards the three species than their counterparts derived from auxin derivative **15** (**23** and **24**). All these compounds were more active towards *P. ramosa* than to the other two species, especially compounds **23** and **24** that were not active towards *O. minor*, and hybrid **24** that was not active towards *S. hermonthica*. Xu *et al*. recently described a clade of ShHTL receptors from *S. hermonthica* hyposensitive to light that bind SLs with diverse affinity.^45^ Some of our new hybrid‐type compounds seem to be species selective, namely **23** and **24**. This could be a reason for the different levels of ShHTL receptor expression among species.

We showed that D‐ring modification is important for SL perception. Double methylation of the D‐ring caused a decrease in bioactivity and triple methylation led to total loss of bioactivity. This result is relevant to the previously published mode of action.^2^, ^46^ However, it does not correlate with the results obtained on GR24 and Nijmegen‐1 modified at C‐2.^40^ Possibly, the ethyl ester auxin moiety plays a role in affecting the bioactivity.

Several articles analysing the seed germination inducing activity of SL analogues have been published. Jamil *et al*. published a remarkable result, where the half maximal concentration of a methyl phenlactonoate (MP1) was 1 nm towards *S. hermonthica* and the activity on *P. ramosa* was similar to control.^47^ Saccharin and cyclic keto–enol analogues were moderately active towards *S. hermonthica* and comparable with **18** and **22**.^31^, ^48^ Fluorescent analogues are worth mentioning, although tested on a different parasitic weed (*Orobanche aegyptiaca*), which showed bioactivity at picomolar levels.^49^, ^50^ However, no such high activity was observed for *P. ramosa* and *O. minor* compared with our SL analogues **18** and **22**, which are outstandingly active. Boyer *et al*. published 3′‐methyl‐GR24 having an EC_50_ value one order of magnitude higher than that of GR24 on *P. ramosa* and comparable with GR24 for *O. minor*.^51^ Several hydroxylated analogues of GR24 showed germination‐stimulating activity at 0.1 μm, but results at lower concentrations were not tested.^52^

The new hybrid molecules **18** and **22** elicited the germination of seeds of parasitic weeds to a significant extent. Considering a suicidal germination approach, they could be agronomically interesting in terms of depleting the parasitic seed bank in soils.^24^, ^53^

## 4 CONCLUSION

In conclusion, new hybrid‐type SL analogues of auxin derivative ethyl 2‐(1*H*‐indol‐3‐yl)acetate and of ethyl 2‐phenylacetate showed considerable germination activity on parasitic weed seeds. Some of these analogues, namely compounds **18** and **22** (with the monomethylated natural D‐ring), were the most effective germination stimulants, with EC_50_ values in the nm range. Activity was strongly influenced by the incorporation of methyl groups on the D‐ring, with the trimethylated derivatives presenting no activity. These new analogues are of potential interest for agronomic applications, e.g. for the control of parasitic weeds, because of their ease of preparation and relevant activity.

## Supporting information


**Appendix S1** Supporting information
