# Supplementary material for: Hybrid‐type strigolactone analogues derived from auxins
Source: Pest Manag Sci. 2019 Aug 23;75(11):3113–21. doi: 10.1002/ps.5553 (PMC6852101; doi:10.1002/ps.5553)
Supplement: Supplementary file 1 — Appendix S1 Supporting information [file PS-75-3113-s001.docx]

Supporting information

**Hybrid-Type Strigolactone Analogues Derived from Auxins**

Daniel Blanco-Ania*^a^, Jurgen J. Mateman^a^, Adéla Hýlová^b^, Lukáš Spíchal^b^, Luc M. Debie^a^ and Binne Zwanenburg*^a,b^

^a^Radboud University, Institute for Molecules and Materials, Cluster of Organic Chemistry, Heyendaalsweg 135, 6525AJ Nijmegen, the Netherlands

^b^Palacký University, Faculty of Science, Centre of the Region Haná for Biotechnological and Agricultural Research, Department of Chemical Biology and Genetics, Slechtitelu 241/27, CZ-783 71 Olomouc, Czech Republic

**^1^H and ^13^C NMR spectra of compounds** **5–12 and 16–25** S2–S33

^1^H NMR Compound **5**

^1^H NMR Compound **6**

^13^C NMR Compound **6**

^1^H NMR Compound **7**

^1^H NMR Compound **8**

^1^H NMR Compound **9**

^13^C NMR Compound **9**

^1^H NMR Compound **10**

^13^C NMR Compound **10**

^1^H NMR Compound **11**

^13^C NMR Compound **11**

^1^H NMR Compound **12**

^13^C NMR Compound **12**

^1^H NMR Compound **16**

^1^H NMR Compound **17**

^13^C NMR Compound **17**

^1^H NMR Compound **18**

^13^C NMR Compound **18**

^1^H NMR Compound **19**

^13^C NMR Compound **19**

^1^H NMR Compound **20**

^13^C NMR Compound **20**

^1^H NMR Compound **21**

^13^C NMR Compound **21**

^1^H NMR Compound **22**

^13^C NMR Compound **22**

****^1^H NMR Compound **23**

^13^C NMR Compound **23**

^1^H NMR Compound **24**

^13^C NMR Compound **24**

^1^H NMR Compound **25**

^13^C NMR Compound **25**
